# A wide spectrum of clinical and brain MRI findings in patients with *SLC19A3 *mutations

**DOI:** 10.1186/1471-2350-11-171

**Published:** 2010-12-22

**Authors:** Kenichiro Yamada, Kiyokuni Miura, Kenju Hara, Motomasa Suzuki, Keiko Nakanishi, Toshiyuki Kumagai, Naoko Ishihara, Yasukazu Yamada, Ryozo Kuwano, Shoji Tsuji, Nobuaki Wakamatsu

**Affiliations:** 1 Department of Genetics, Institute for Developmental Research, Aichi Human Service Center, Aichi, Japan; 2 Department of Pediatric Neurology, Central Hospital, Aichi Human Service Center, Aichi, Japan; 3 Department of Neurology, Brain Research Institute, Niigata University, Niigata, Japan; 4 Department of Perinatology, Institute for Developmental Research, Aichi Human Service Center, Aichi, Japan; 5 Department of Molecular Genetics, Brain Research Institute, Niigata University, Niigata, Japan; 6 Department of Neurology, University of Tokyo, Tokyo, Japan

## Abstract

**Background:**

SLC19A3 (solute carrier family 19, member 3) is a thiamin transporter with 12 transmembrane domains. Homozygous or compound heterozygous mutations in *SLC19A3 *cause two distinct clinical phenotypes, biotin-responsive basal ganglia disease and Wernicke's-like encephalopathy. Biotin and/or thiamin are effective therapies for both diseases.

**Methods:**

We conducted on the detailed clinical, brain MRI and molecular genetic analysis of four Japanese patients in a Japanese pedigree who presented with epileptic spasms in early infancy, severe psychomotor retardation, and characteristic brain MRI findings of progressive brain atrophy and bilateral thalami and basal ganglia lesions.

**Results:**

Genome-wide linkage analysis revealed a disease locus at chromosome 2q35-37, which enabled identification of the causative mutation in the gene *SLC19A3*. A pathogenic homozygous mutation (c.958G > C, [p.E320Q]) in *SLC19A3 *was identified in all four patients and their parents were heterozygous for the mutation. Administration of a high dose of biotin for one year improved neither the neurological symptoms nor the brain MRI findings in one patient.

**Conclusion:**

Our cases broaden the phenotypic spectrum of disorders associated with *SLC19A3 *mutations and highlight the potential benefit of biotin and/or thiamin treatments and the need to assess the clinical efficacy of these treatments.

## Background

The *SLC19 *(solute carrier family 19) gene family (comprising *SLC19A1, SLC19A2*, and *SLC19A3*) is responsible for the uptake of water-soluble vitamins into cells. *SLC19A1 *was first identified as a gene that could restore reduced folate carrier activity and methotrexate sensitivity in methotrexate-resistant human breast cancer cells [1]. *SLC19A2 *was isolated as the gene responsible for thiamin-responsive megaloblastic anemia syndrome (TRMA; MIM 249270) [2-4] and was identified as a thiamin transporter [5]. *SLC19A3 *was cloned based on its homology with *SLC19A1 *and *SLC19A2*, and it was identified as a second thiamin transporter [6-8]. There are significant structural similarities among the three SLC19 proteins at the amino-acid-sequence level (> 39% identity). All three *SLC19 *genes encode transporter proteins composed of 12 transmembrane domains, and all are expressed ubiquitously [9]. Two apparently unrelated autosomal recessive disease phenotypes, biotin-responsive basal ganglia disease (BBGD; MIM 607483) [10,11] and Wernicke's-like encephalopathy (MIM 606152) [12], are associated with mutations of *SLC19A3*. We herein report on a study of four patients that presented with epileptic spasms in early infancy, severe psychomotor retardation, and characteristic brain MRI findings of progressive brain atrophy and bilateral thalami and basal ganglia lesions. We found that these phenotypes were caused by a homozygous *SLC19A3 *mutation and discuss the phenotype-genotype correlation of the disorders associated with *SLC19A3 *mutations.

## Methods

### Case Presentation

The experiments were conducted after approval by the institutional review boards at the Institute for Developmental Research, Aichi Human Service Center and Brain Research Institute, Niigata University. Written informed consent was obtained from the parents of the four patients participating in this study.

### Patient V-2

**Figure 1:**
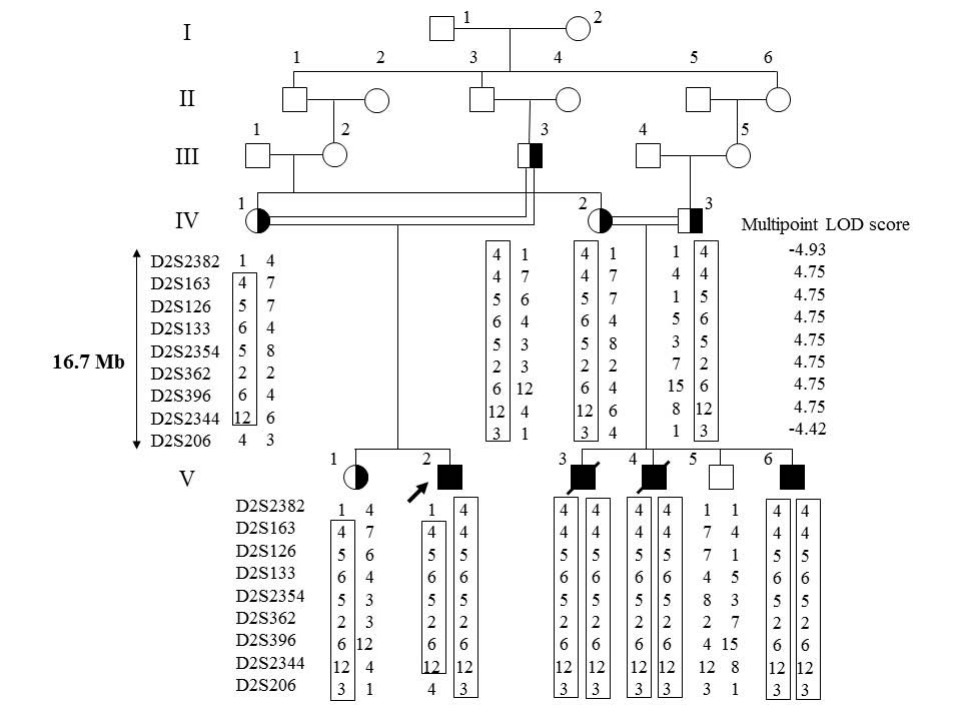
**Pedigree and haplotypes in the linkage region**. Squares and circles indicate males and females, respectively. The proband and affected individuals are indicated by an arrow and closed symbols, respectively. Only informative markers that define the genetic breakpoints are shown. The maximum LOD score was in the region between *D2S163 *to *D2S2344*.

**Figure 2:**
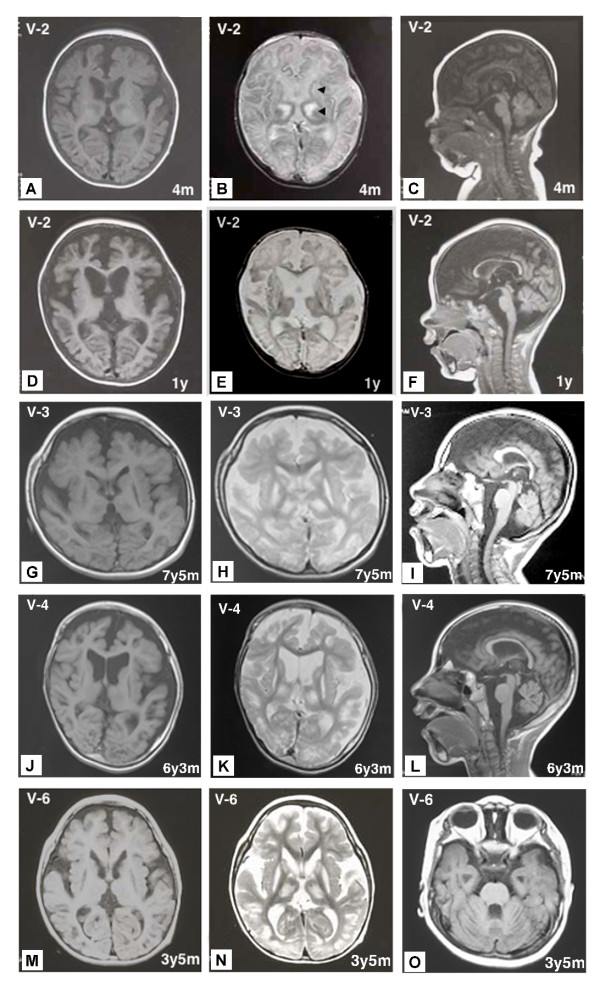
**MRIs of patients V-2, V-3, V-4 and V-6 at various ages**. Areas of abnormal intensity in the thalami and basal ganglia (arrowheads) were distinct at 4 months in V-2 (A, B). The sagittal and axial views of T1- and T2-weighted images of V-2 at 1 year showed severe brain atrophy (D, E, F). The sagittal and axial views of T1- and T2-weighted images of V-3 at 7 years, 5 months (G, H, I), V-4 at 6 years, 3 months (J, K, L), and V-6 at 3 years, 5 months (M, N, O) showed severe brain atrophy, including cerebellar atrophy (I, L, O) and abnormal intensity areas in the thalami and basal ganglia.

The proband was an 18-year-old Japanese boy born to healthy consanguineous parents (Figure 1): his maternal grandmother and father are first cousins. Notably, his three cousins (Figure 1 patients V-3, V-4, and V-6) had very similar clinical courses and features. This patient was born at a gestational age of 38 weeks without complications. At 1 month of age, he presented an opisthotonic posture, and at 2.5 months of age, epileptic spasms appeared. His EEG demonstrated multifocal spikes but no hypsarrhythmia; thus, he was diagnosed with atypical infantile spasms without hypsarrhythmia. A complete blood count, biochemical analyses for a wide range of metabolic disorders and an immunohistochemical analysis of skeletal muscle biopsies were normal. Two courses of ACTH therapy were performed but were only transiently effective. At 1 year of age, intractable tonic seizures appeared, and he showed severe mental retardation and spastic quadriplegia with an increased jaw-jerk reflex and hyperreflexia in the extremities, including positive Babinski sign. Involuntary movement was not observed. Brain MRI at 4 months of age revealed symmetrical abnormal intensities in the bilateral thalami and basal ganglia on T1- and T2-weighted images (Figure 2A and 2B). Profound brain atrophy progressed at 1 year (Figure 2C, 2D, 2E and 2F). At 12 years of age, gastrostomy was performed because of swallowing difficulties. At present, he is bedridden with contractures of the extremities and has several tonic seizures daily.

### Patient V-3 and Patient V-4

Two other patients who are cousins of patient V-2 and born to parents who are second cousins also had quite similar clinical course and brain MRI findings (Figure 2G, H, I, J, K, L and Table 1). Patients V-3 and V-4 died of respiratory failure at 12 years and 3 months and 9 years and 7 months of age, respectively.

**Table 1:** Clinical and brain MRI findings of the present patients, BBGD and Wernicke's-like encephalopathy caused by *SLC19A3* mutations

Patients		V-2	V-3	V-4	V-6	BBGD	Wernicke's-like
Present age		18Y	12Y(dead)	9Y(dead)	6Y	9-33Y	ND

Sex		male	male	male	male	male, female	male

Consanguinity		+	+	+	+	+/-	-

Neurological findings							

	bad temper	1M	2M	2M	2M	ND	ND
	head control	-	-	-	-	+	+
	opisthotonic posture	1M	-	2M	11M	++	-
	mental retardation	++	++	++	++	++ (without treatment)	-
	dysphagia	++	++	++	+	+	-
	pyramidal signs	++	++	++	++	++	-
	quadriplegia	++	++	++	++	++	-
	dystonia	-	-	-	-	++	-
	cogwheel rigidity	-	-	-	-	++	-
	ophthalmoplegia	-	-	-	-	+	++
	nystagmus	-	-	-	-	-	++
	ataxia	-	-	-	-	-	++
Seizure							
	onset	2M	3M	2M	11M	childhood	second decade
	type	ES→SGE	ES→SGE	ES→SGE	ES→PE	PE, SGE	PE

Gastrostomy		12Y	7Y	6Y	-	-	-
Tracheotomy		-	4Y	4Y	-	-	-

Pathologic brain MRI findings		11M	7Y	6Y	14M	3-33Y	36Y

	cerebral atrophy	++	++	++	++	-	-
	cerebellar atrophy	++	+	++	+	-	-
	brain stem atrophy	+	-	-	-	-	-
Abnormal signals in							
	thalamus	+	+	+	+	-	+
	caudate nuclei	+	+	+	+	+	-
	putamen	+	+	+	+	+	-
	cortical & subcortical region	-	-	-	-	+	-
	periaqueductal region	-	-	-	-	-	+

Treatment		NP	NP	NP	biotin: ineffective	biotin (and thiamin): effective	thiamin: effective

Y = year(s); M = month(s); ES = epileptic spasm; SGE = symptomatic generalized epilepsy; PE = partial epilepsy; (+) = presence of clinical signs, + to ++ increasing severity; (-) = minus sign; ND = not described; NP = not performed. Typical clinical and brain MRI findings of BBGD [10,16] and Wernicke's-like encephalopathy [12] are summarized.

### Patinet V-6

Patient V-6 was born at 40 weeks gestation without complications. He is the fourth child and the younger brother of patients V-3 and V-4. At 11 months of age, he began experiencing epileptic spasms in clusters. His EEG demonstrated anterior dominant irregular diffuse spikes and waves, and polyspikes and waves; however, hypsarrhythmia was not observed. He was diagnosed with atypical infantile spasms. At 1 year of age, he was admitted to our hospital for treatment for epilepsy. He was able to eat weaning food; however, he did not exhibit head control and was unable to roll over. Neurological examination revealed generalized hypotonia, and deep tendon reflexes of the extremities were not elevated. ACTH therapy was transiently effective in alleviating the attacks. Brain MRI at 14 months of age demonstrated severe cortical atrophy and abnormal intensities in the bilateral thalami and basal ganglia. Physical examination at 2 years of age demonstrated severe psychomotor retardation; he was not able to sit without support and was also unable to roll over. Neurological examination demonstrated generalized hypotonia with spasticity of limbs and severe mental retardation. Involuntary movement was not observed. Because he was identified as homozygous for a *SLC19A3 *mutation, patient V-6 had taken biotin (5 mg/kg/day) for one year beginning at 19 months of age with informed consent from his parents; however, his neurological symptoms and brain MRI findings did not improve. Brain MRI at 3 years and 5 months of age demonstrated diffuse brain atrophy and abnormal intensities in the bilateral thalami, caudate nuclei and putamen on T1- and T2-weighted images (Figure 2M, 2N and 2O). Currently, Patient V-6, at 6 years of age, is bedridden. The clinical features and brain MRI findings of the four patients are summarized in Table 1.

### Genome-Wide Linkage Analysis

We performed a genome-wide linkage analysis in ten family members related to the four neurological patients using 763 microsatellite markers (Applied Biosystems, Foster City, CA) to cover the all autosomes with an average interval of 4.6 cM. Multi-point LOD (logarithm of odds) scores were calculated using the Allegro program for all loci under the assumption of autosomal recessive inheritance and a disease frequency of 0.001 [13]. Haplotypes were constructed manually to minimize the number of recombination events.

### Genetic Analyses

Genomic DNA was isolated from white blood cells by phenol/chloroform extraction. PCR-amplified DNA fragments were isolated with a QIAEX II Gel Extraction Kit (QIAGEN, Valencia, CA) and purified using polyethylene glycol (PEG 6000) precipitation. Isolated PCR products were cloned and sequenced with an SQ5500E DNA sequencer (HITACHI, Tokyo, Japan), or were sequenced directly with specific inner primers or the same primers used for PCR [14].

### Construction of Expression Vectors

First-strand cDNA was synthesized by reverse transcription of 10 μg of total RNA from normal or the proband's lymphoblastoid cells using a First-Strand cDNA Synthesis Kit (GE Healthcare, Tokyo, Japan) with specific antisense primer (A1: 5'-GTTGCGTCTAGATTAGAGTTTTGTTGAC-3', *Xba*I site is underlined) in a reaction volume of 15 μl. The cDNA products were amplified with LA-Taq DNA Polymerase (TAKARA BIO INC, Otsu, Japan) using the specific primers (S1: 5'-AACAGACACTCCCTTCTGAATTCATG-3' and A1, *Eco*RI site is underlined). PCR was performed in a total volume of 20 μl for 36 cycles as follows: denaturation at 94°C for 30 s, annealing at 58°C for 30 s, and extension at 72°C for 90 s. The 1,535-bp PCR products were subcloned into the *Eco*RI/*Xba*I sites of the pCI-neo vector, a mammalian expression vector driven by a cytomegalovirus (CMV) promoter and enhancer, to generate pCI-neo-SLC19A3(WT) and pCI-neo-SLC19A3(E320Q).

### Thiamin Transport Assay

We performed a thiamin transport assay according to the protocol of Ashokkumar *et al*. [15]. Briefly, a 1.0 μg aliquot of the *SLC19A3 *expression vector (pCI-neo-SLC19A3(WT), pCI-neo-SLC19A3(E320Q)) and the control vector (pCI-neo) were transfected into HEK293 cells in 24-well plates using Lipofectamine 2000 Reagent (Invitrogen, Carlsbad, CA). Two days after transfection, the HEK293 cells were washed two times with pre-warmed phosphate-buffered saline (PBS) and incubated for 10 min at 37°C in 200 μl of assay buffer (20 mM HEPES (pH 7.4), 140 mM NaCl, 5 mM KCl, 2 mM MgCl_2_, and 5 mM sucrose) containing 0.1 μM of [^3^H]-labeled thiamin (14.8 kBq:0.74TBq/mmol) with or without 0.1 mM unlabeled thiamin. Thiamin transport was terminated by the addition of 0.5 ml of ice-cold PBS followed by immediate aspiration. Cells were rinsed two times with 0.5 ml of ice-cold PBS and lysed with 200 μl of 0.2 M NaOH for 30 min at 65°C. A 50-μl aliquot of lysate was spotted onto Whatman 3 MM chromatography paper (GE Healthcare), and radioactivity counts were conducted using a liquid scintillation counter. Protein concentrations were estimated using an Advanced Protein Assay Kit (Cytoskeleton Inc. Denver, CO) according to the manufacturer's instructions.

## Results

### The Disease Maps to Chromosome 2q35-37

The patients' characteristic clinical features without obvious extrapyramidal symptoms, rapid clinical course and brain MRI findings did not lead us to diagnosis a specific disease. However, we conducted a genome-wide linkage analysis that revealed a disease locus in the region from *D2S2382 *to *D2S206 *on 2q35-37 (~16.7 Mb) with a maximum multipoint LOD score of 4.75 (Figure 1).

### Identification of Homozygous *SLC19A3 *Mutation

**Figure 3:**
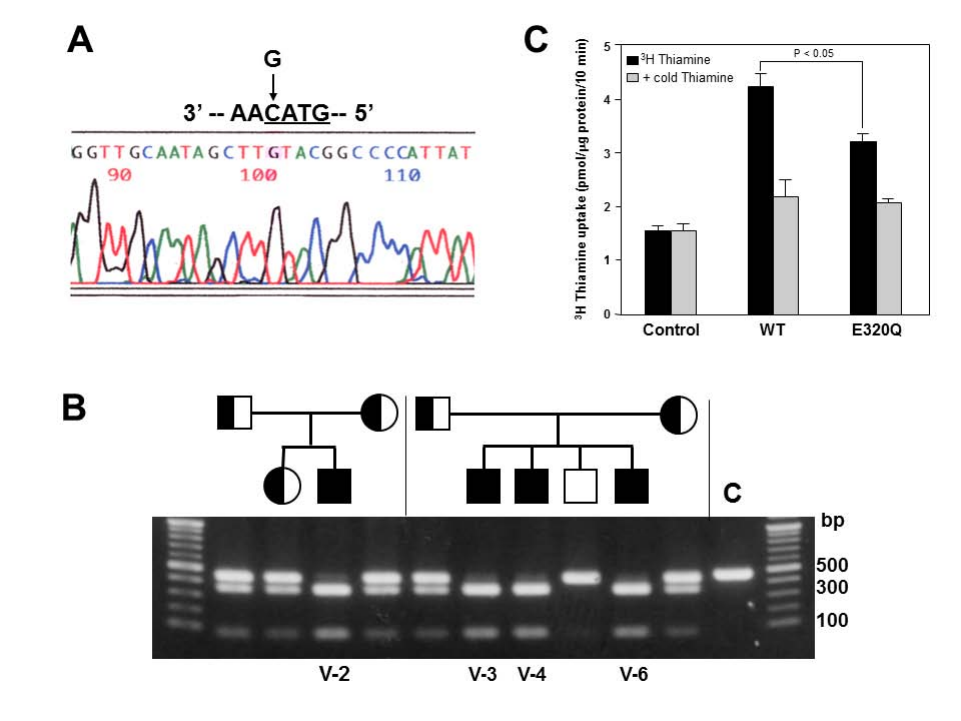
**Identification and characterization of a disease mutation (c.958G > C, [p.E320Q]).** (A) The sequence analyses showed a G to C substitution at nucleotide position 958 in Exon 3 of *SLC19A3*, resulting in a substitution of glutamic acid at codon 320 for glutamine (c.958G > C, [p.E320Q]), indicated by an arrow. (B) *Rsa*I (GTAC)-digested PCR products were run through a 1.2% agarose gel. Digestion of products containing the E320Q mutation resulted in 357- and 86-bp fragments. All patients (V-2, V-3, V-4 and V-6) were homozygous for the mutation, and their parents were heterozygous for the mutation. (C) [^3^H]-thiamin uptake by HEK293 cells expressing wild-type or E320Q SLC19A3. Two days after transfection, cells were incubated with 0.1 μM of [^3^H]-labeled thiamin (14.8 kBq:0.74TBq/mmol) for 10 min with or without 0.1 mM unlabeled thiamin. Incorporated [^3^H]-thiamin was measured using a liquid scintillation counter. Results are expressed as mean ± SE of specific uptake values from three independent experiments.

*SLC19A3 *gene maps in the region, and then we identified a homozygous mutation (c.958G > C, [p.E320Q]) in exon 3 of *SLC19A3 *in all patients, while their parents were heterozygous for the mutation (Figure 3A and 3B).

### Determination of thiamin transport activity of mutant SLC19A3 Proteins in HEK293 cells

To address the effect of the E320Q mutation on thiamin transport activity, we transfected wild-type and mutant SLC19A3 expression constructs into HEK293 cells. We then analyzed the uptake of [^3^H]-labeled thiamin into these cells. We found that the E320Q mutation decreased thiamin uptake to 63% of that of the wild-type protein (Figure 3C), corroborating the findings of Kono *et al*. [12] using Chinese hamster ovary (CHO) cells.

## Discussion

In the present study, we demonstrated that a homozygous mutation (E320Q) in *SLC19A3 *is associated with epileptic spasms in early infancy, severe psychomotor retardation and characteristic MRI findings, including progressive brain atrophy and bilateral thalami and basal ganglia lesions. It should be noted that four patients in this family showed quite similar clinical presentations, strongly arguing for the distinct genotype-phenotype correlation associated with the homozygous E320Q mutation. Two apparently unrelated disease phenotypes, BBGD (MIM 607483) [10,11] and Wernicke's-like encephalopathy (MIM 606152) [12], have been shown to be associated with mutations of *SLC19A3*. Patients of BBGD with homozygous mutations (G23V, T422A) in *SLC19A3 *showed childhood-onset encephalopathy, characterized by epilepsy, confusion, external ophthalmoplegia, dysarthria, dysphagia, dystonia, rigidity and quadriparesis. In these patients, the administration of high doses of biotin (5-10 mg/kg/day) early in the progression of the disorder eliminated their symptoms, whereas thiamin was ineffective [10,11]. Recently, compound heterozygous mutations (E320Q, K44E) in *SLC19A3 *were shown to cause Wernicke's-like encephalopathy, which is characterized by acute onset of epilepsy, ataxia, nystagmus and ophthalmoplegia in the second decade of life, and symptoms are effectively alleviated with thiamin treatment [12].

Despite sharing one mutant allele (E320Q) with the known causative mutations in Wernicke's-like encephalopathy, our patients clearly differed in age at onset, brain MRI findings and symptoms, raising the possibility that the functions altered by the mutant *SLC19A3 *with K44E are substantially different (Table 1). Although both the BBGD phenotype and our patients showed similar bilateral basal ganglia lesions in the brain and encephalopathy that are fatal unless treated, the clinical presentations of our patients are distinct from the previously reported BBGD phenotype in the following ways: (1) epileptic spasms appeared in infancy in all of our patients, in contrast to the epilepsy onset in childhood for patients with BBGD; (2) MRI findings in our patients are characterized by progressive brain atrophy and additional lesions in the bilateral thalami that are not observed in patients with BBGD; (3) our patients did not display obvious dystonia and cogwheel rigidity reflecting dysfunctions in basal ganglia; (4) administration of a high dose of biotin for one year improved neither the neurological symptoms nor the brain MRI findings in Patient V-6, though it is unclear whether administration of biotin at early stages of the disorder (e.g., when epileptic spasms first appeared) improved the subsequent clinical trajectory (Table 1). Thus our cases broaden the phenotypic spectrum of disorders associated with mutations in *SLC19A3*.

Recently, two novel mutations, both of which created premature stop codons, were identified in BBGD patients [16]. Transfection studies demonstrated that G23V and T422A mutations identified in the BBGD phenotype lead to mutant SLC19A3 that is nonfunctional for thiamin uptake activity [8]. These findings suggest that loss-of-function mutations in SLC19A3 are associated with the BBGD phenotype. In contrast to these loss-of-function mutations, we demonstrated that mutant SLC19A3 with E320Q possesses some thiamin uptake activity that is approximately 60% of wild-type SLC19A3 in CHO cells [12] and HEK293 cells (Figure 3C). These findings alone seem difficult to account for severe clinical presentations in our cases. Interestingly, a previous report showed that the negative charge at position E320 was conserved in other transporters (SLC19A1, SLC19A2) and was crucial for the formation of a salt bridge with a conserved, positively-charged residue, K380 [8]. The homozygous mutation of E320 may, therefore, destabilize the protein conformation and bring with more deleterious effect, including gain-of-toxic function and/or the dramatically changed protein structure.

In this study, the efficacy of high doses of biotin and/or thiamin for the clinical phenotypes of our patients has not been determined. This indicates that we have not determined whether our patients are phenotypic variation of "biotin-responsive" basal ganglia disease or "biotin-unresponsive" another type of a disease. Therefore, accumulation of genetic analysis and clinical courses from more patients who undergo biotin and/or thiamin treatment as well as further laboratory studies employing cellular or mouse models will be needed to better characterize the clinical phenotypes and biochemical characteristics of the homozygous E320Q mutation in *SLC19A3*.

## Conclusions

The present study indicated that four Japanese patients in a Japanese pedigree who presented with epileptic spasms in early infancy, severe psychomotor retardation, and characteristic brain MRI findings of progressive brain atrophy and bilateral thalami and basal ganglia lesions were caused by homozygous mutation (c.958G > C, [p.E320Q]) in *SLC19A3*. Our cases broaden the phenotypic spectrum of disorders associated with *SLC19A3 *mutations and highlight the potential benefit of biotin and/or thiamin treatments and the need to assess the clinical efficacy of these treatments.

## Abbreviations

MRI: Magnetic Resonance Imaging; EEG: Electroencephalogram; ACTH: Adrenocorticotropic hormone

## Competing interests

The authors declare that they have no competing interests.

## Authors' contributions

KY participated in planning and design of the study, performed gene sequencing analysis and interpretation of the data and drafted and finalized the manuscript. KM, MS, TK, NI examined the clinical features of each patients and contributed to acquisition of materials, interpretation of data and drafted the manuscript. KH and RK performed genome-wide linkage analysis and contributed to interpretation of the data and drafted the manuscript. KN performed transient expression study of wild-type and mutant SLC19A3 proteins and contributed to interpretation of data and drafted the manuscript. YY performed gene sequencing analysis and interpretation of the data and drafted the manuscript. ST conducted genome-wide linkage analysis and contributed to interpretation of data, drafted and finalized the manuscript. NW organized this study, performed interpretation of the data and drafted and finalized the manuscript. All authors read and approved the final manuscript.

## Pre-publication history

The pre-publication history for this paper can be accessed here:

http://www.biomedcentral.com/1471-2350/11/171/prepub
